# Enhanced Melt Memory Effects in Poly(butylene succinate) Through Incorporation of Extended-Chain Crystals

**DOI:** 10.3390/polym17081086

**Published:** 2025-04-17

**Authors:** Xue-Wei Wei, Jun Xu, Jia-Yao Chen, Bao-Hua Guo, Hai-Mu Ye

**Affiliations:** 1 Institute of Polymer Science & Engineering, Department of Chemical Engineering, Tsinghua University, Beijing 100084, China; wxw8383@163.com (X.-W.W.); jun-xu@mail.tsinghua.edu.cn (J.X.); 2 Department of Materials Science and Engineering, College of New Energy and Materials, China University of Petroleum, Beijing 102249, China; 2021310610@student.cup.edu.cn

**Keywords:** extended-chain crystals, nucleating agents, self-nucleation, poly(butylene succinate), rheological properties

## Abstract

Extended-chain crystals (ECCs) of poly(butylene succinate) (PBS), featuring highly ordered molecular chains and exceptional thermal stability with a melting point 25 °C higher than conventional folded-chain crystals, demonstrate remarkable potential as nucleating agents for PBS crystallization. The incorporation of 5 wt% ECCs leads to a 12 °C increase in crystallization temperature and reduces crystallization half-time by 88% at 98 °C. Most significantly, self-nucleation studies reveal an unprecedented expansion of *Domain II* temperature window (∆*T* = 30 °C), exhibiting enhanced melt memory effects and a broadened processing window. Rheological investigations uncover the formation of unique intermolecular interactions in the melt, evidenced by non-terminal viscoelastic behavior and reduced loss tangent at elevated temperatures, with complementary evidence from higher ECC loadings confirming these findings. This local ordered structure exhibits remarkable thermal stability, persisting even above the melting point through specific intermolecular interactions, leading to the melt memory effect. These findings establish a new paradigm for nucleation control in biodegradable polyesters and provide molecular design strategies for high-performance sustainable materials.

## 1. Introduction

The growing environmental concerns have intensified the demand for sustainable polymeric materials, particularly biodegradable polymers as alternatives to conventional plastics [1,2,3]. Among various biodegradable polymers, poly(butylene succinate) (PBS) has garnered significant attention due to its unique combination of biodegradability, and thermoplastic characteristics [4,5]. Despite its promising characteristics, high-performance applications of PBS remain constrained by its slow crystallization kinetics [6], which significantly influences both processing efficiency and final material properties [7,8,9]. Additionally, serious post-crystallization causes dimensional instability and mechanical deterioration, further limiting its long-term practical applications [10].

The incorporation of heterogeneous nucleating agents represents the most direct and industrially viable approach for enhancing PBS crystallization rate. Traditional nucleating agents, including inorganic nanoparticles and organic additives, function primarily by reducing the surface free energy barrier for nucleus formation [11,12,13,14]. However, these nucleating agents often suffer from poor dispersion and incompatibility with the matrix [15,16], potentially compromising the material’s mechanical properties and optical properties. Recent advances in nucleation control have highlighted the critical role of epitaxial nucleation in determining nucleation efficiency [17,18,19,20]. This understanding has led to the development of macromolecular nucleating agents that exploit structural similarity with the PBS crystal lattice. For instance, poly(butylene succinate-*co*-butylene fumarate) (PBSF) and poly(butylene fumarate) (PBF) demonstrate enhanced nucleation through isomorphic crystallization mechanisms [21,22,23,24]. They not only exploit structural similarity for epitaxial nucleation but also leverage the elevated melting temperatures (typically 20 °C higher than PBS) to create a thermal processing window. However, the precise molecular design requirements and the cell structure matching for the polymeric nucleating agents have limited their practical application. Previous studies have demonstrated the effectiveness of ordered structures in polymer crystallization. Karger-Kocsis revealed how shear-induced precursors enhance nucleation [25], while Xu et al. established the concept of “cloning” polymer crystals through self-seeding [26]. Our approach extends these concepts using highly ordered extended-chain crystals with PBS matrix compatibility.

Extended-chain crystals (ECCs) of PBS have emerged as an alternative for nucleation control, offering unique advantages in thermal stability and structural compatibility [27]. Unlike conventional folded-chain crystals (FCCs), ECCs are characterized by fully extended polymer chains and exceptionally high structural order, resulting in significantly elevated melting temperatures (Δ*T*_m_ ≈ 25 °C higher than conventional PBS crystals) and enhanced crystallinity [28]. This remarkable thermal stability allows ECC to maintain its crystalline structure at temperatures well above the normal processing range of PBS, potentially overcoming the limitations of traditional nucleating strategies. The concept finds parallels in the stereocomplex crystals (SCs) formed between poly(*L*-lactide) (PLLA) and poly(*D*-lactide) (PDLA) [29,30,31], which demonstrate exceptional nucleation efficiency due to their elevated melting temperature (*T*_m_ ≈ 230 °C, approximately 50 °C higher than PLLA homocrystals). Notably, SCs have been shown to form physical network structures in the polymer melt, significantly enhancing the crystallization kinetics and regulating rheological properties [32]. The addition of merely 0.25 wt% PDLA can increase nucleation density by a factor of 150, far exceeding the effect of traditional nucleating agents [33].

Inspired by these advances, we present a comprehensive investigation of ECC as the nucleating agent for PBS crystallization. Our study employs a novel analytical approach combining self-nucleation studies [34,35,36,37,38] with rheological analysis to elucidate the formation and evolution of ECC-induced ordered structures. Particular emphasis is placed on understanding the complex interplay between thermal history, ordered structures in melt, and nucleation efficiency, providing fundamental insights into the design of nucleating agents for high-performance sustainable polymers.

## 2. Experimental

### 2.1. Materials

Commercial poly(butylene succinate) (PBS) with a viscosity-average molecular weight (*M*_η_) of 90.0 kg·mol^−1^ was obtained from Xinjiang Blue Ridge Tunhe Science & Technology Co., Ltd. (Changji, China). The as-received PBS exhibited a melting temperature of 113.5 °C. Urea (AR grade), chloroform (CHCl_3_, AR grade), dichloromethane (CH_2_Cl_2_, AR grade), and methanol (CH_3_OH, AR grade) were purchased from Shanghai Aladdin Company (Shanghai, China). All the chemical reagents were used as received without further purification.

### 2.2. Preparation of PBS/Urea Complex

The PBS/urea complex was fabricated via a controlled semi-solid phase blending process using a Brabender mixer Kneter-W50-EHT (Brabender GmbH & Co. KG, Duisburg, Germany). Prior to this process, both PBS and urea were thoroughly dried in a vacuum oven at 60 °C for 12 h to remove the residual water. The blending process was conducted through an optimized sequential feeding procedure. Initially, PBS was processed at 125 °C with a screw speed of 120 rpm for 5 min to achieve uniform melting while minimizing thermal degradation. Subsequently, urea was introduced at a PBS/urea mass ratio of 1:1.75, followed by continued mixing for an additional 10 min to ensure homogeneous distribution.

### 2.3. Extraction of Extended-Chain Crystals

Extended-Chain Crystals (ECCs) were extracted from the PBS/urea complex through a dissolution process at room temperature. The complex was immersed in deionized water at a blend-to-water ratio of 1:10 (*w*/*w*) and subjected to gentle stirring for water-washing treatment. To ensure the complete removal of urea, the washing water was replaced with fresh deionized water every 12 h. This water-exchange process was continued until the complete elimination of urea residues. The resulting precipitate was collected via filtration and subsequently dried under vacuum conditions at 60 °C for 24 h to obtain the purified ECCs.

### 2.4. Preparation of PBS/ECCs Composites

PBS/ECCs composites were prepared through a solution mixing process using dichloromethane (CH_2_Cl_2_) as the selective solvent, which can dissolve PBS but not ECC. This selective solubility enables the uniform dispersion of ECCs in the PBS matrix while maintaining its extended-chain crystal structure. Two compositions with ECC contents of 1, 5, and 10 wt% were prepared, denoted as PBS/ECCs-1, PBS/ECCs-5, and PBS/ECCs-10, respectively. For each composition, PBS was first dissolved in CH_2_Cl_2_ to form a 10 wt% solution, followed by the addition of predetermined amounts of ECCs. The mixtures were subjected to continuous stirring at room temperature for 3 h to achieve uniform dispersion. Subsequently, the solvent was evaporated at 60 °C under atmospheric pressure, and the resulting composites were further dried under vacuum at 60 °C for 12 h to ensure complete removal of residual solvent.

### 2.5. Characterization

Molecular Weight Analysis: Intrinsic viscosity measurements were conducted using an Ubbelohde viscometer at 25 °C. Samples were dissolved in a phenol-tetrachloroethane mixture (1:1 by mass) at a concentration of 0.005 g/mL. The viscosity-average molecular weight (*M*_η_) was determined using the Solomon-Ciuta method and the Mark–Houwink parameters calibrated for PBS (*K* = 1.7602 × 10^−4^ and α = 0.7883) [39,40,41].

Crystal structural Analysis: Crystal structure was determined using Rigaku 3 kW SmartLab Wide angle X-ray diffractometer (WAXD, Rigaku Corporation, Tokyo, Japan) with Cu K*α* radiation (λ = 0.154 nm). Samples were prepared as uniform films to ensure consistent X-ray penetration depth. Diffraction patterns were collected at room temperature (25 °C) over a 2θ range of 5–40° using a step size of 0.01° and a scanning rate of 10°/min.

Spectroscopic Analysis: Fourier transform infrared spectroscopic measurements were performed using a Bruker Hyperion 2000 spectrometer (FTIR, Bruker Corporation, Ettlingen, Germany). All spectra were acquired with a resolution of 4 cm^−1^ over the wavenumber range of 4000–1000 cm^−1^. Each spectrum represented an average of 32 scans to optimize the signal-to-noise ratio, with data processing conducted using OPUS 7.5 software.

Thermal Analysis: Thermal characteristics were evaluated using a TA Instruments 250 differential scanning calorimeter (DSC, TA Instruments, New Castle, DE, USA) under nitrogen atmosphere (50 mL/min). Thermal analyses were conducted using carefully weighed samples (2.0 ± 0.05 mg) encapsulated in aluminum pans. All thermal protocols began with an initial step by heating to 140 °C (a temperature selected to ensure complete melting of both PBS FCCs and ECCs) and maintaining isothermally for 3 min. Three distinct thermal protocols were employed:

1. Non-isothermal Crystallization Analysis: Samples were cooled to 40 °C at a constant rate of 10 °C/min to investigate the crystallization behavior, followed by reheating to 160 °C at the same rate to examine the melting characteristics. The crystallization temperature (*T*_c_), melting temperature (*T*_m_), and their corresponding enthalpies (Δ*H*_c_, Δ*H*_m_) were determined from the first heating and cooling curves, respectively.
2. Isothermal Crystallization Analysis: Samples were rapidly quenched (50 °C/min) to predetermined crystallization temperatures and maintained isothermally until complete crystallization. The evolution of crystallization was monitored by recording the exothermic heat flow as a function of time.
3. Self-nucleation (SN) investigation: The self-nucleation behavior was investigated using a modified thermal protocol specifically designed to account for the unique structural characteristics of ECCs. Unlike conventional semi-crystalline polymers, ECCs undergo irreversible structural changes upon complete melting, necessitating a carefully tailored three-step thermal procedure (Figure S1 in Supporting Information): (i) The initial step involved controlled heating to a predetermined self-nucleation temperature (*T*_s_) at a rate of 10 °C/min, followed by an isothermal hold period of 5 min. The *T*_s_ values were systematically varied to probe different self-nucleation domains. (ii) Subsequently, samples underwent controlled cooling from *T*_s_ to 40 °C at a constant rate of 10 °C/min. This cooling stage was designed to observe the nucleation efficiency of any remaining self-nuclei or memory effects through the analysis of crystallization temperatures and peak shapes. (iii) The final stage consisted of reheating to 160 °C at 10 °C/min to evaluate the melting behavior of crystals formed during the cooling process. This methodology facilitates quantitative assessment of nucleation efficiency through crystallization temperature shifts and exotherm characteristics. The thermograms revealed three distinct self-nucleation domains characterized by their crystallization behavior: [36,38] *Domain I* exhibiting complete melting with no correlation between *T*_c_ and *T*_s_; *Domain II* displaying prominent self-nucleation effects at intermediate *T*_s_; and *Domain III* manifesting incomplete melting at lower *T*_s_ values.

Morphological Investigation: Spherulitic morphology and the growth process during isothermal crystallization were investigated using an Olympus BX41 polarized optical microscope (POM, Olympus Corporation, Tokyo, Japan) equipped with a Linkam T95-HS temperature-controlled stage and a high-resolution digital camera. Thin film samples (~10 μm) were prepared by melting between two glass cover slides at 140 °C for 3 min, followed by quenched to the desired temperature for isothermal crystallization.

Rheological Measurements: Dynamic rheological characterization was performed using an Anton Paar MCR301 rheometer (Anton Paar GmbH, Graz, Austria) equipped with a parallel-plate geometry (25 mm diameter). Specimens were prepared as disk-shaped samples with dimensions of 25 mm in diameter and 1 mm in thickness. Measurements were conducted within the linear viscoelastic regime using a frequency sweep protocol at multiple isothermal conditions. Prior to each measurement, samples were equilibrated at the test temperature for 5 min to ensure thermal stability and eliminate thermal history effects. Before conducting frequency sweep measurements, strain sweep tests were conducted from 0.01% to 10% strain amplitude at selected frequencies to establish the linear viscoelastic region (LVR) (Figure S2). For each measurement, frequency sweeps were performed over a frequency range of 0.1–100 rad/s at a constant strain of 1%. The complex viscosity (η*), storage modulus (*G*′), and loss modulus (*G*″) were measured at each isothermal condition to evaluate the melt viscoelastic behavior and structural characteristics of the samples. The relaxation modulus (*G*(*t*)) is defined as the ratio of a stress value and an initial strain of the stress relaxation. *G*(*t*) was measured at a strain of 10%, which is in a linear region of all samples at test temperatures.

## 3. Results and Discussion

### 3.1. Structural Characterization

**Figure 1:**
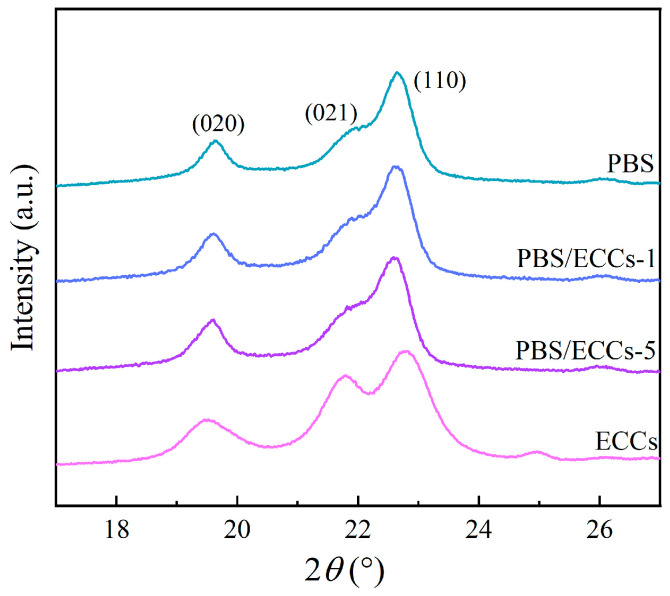
Wide angle X-ray diffractograms for PBS and PBS/ECCs composites after being cooled from melt to 25 °C.

The structural features of PBS and its ECC composites were systematically investigated through WAXD and FTIR spectroscopy. As shown in Figure 1, the WAXD patterns of all samples exhibited characteristic diffraction peaks corresponding to the PBS *α*-form crystal structure [42,43]. For neat PBS, the peaks at 2θ = 19.6°, 21.9°, and 22.6° can be assigned to the (020), (021), and (110) crystallographic planes, respectively [23], consistent with previously reported crystal parameters. The ECCs showed slight peak shifts for these respective planes, while maintaining the fundamental PBS crystal structure. Further crystallographic analysis revealed increased full width at half maximum (FWHM) values for the (020) and (110) reflections in ECCs relative to neat PBS, a phenomenon consistent with previous studies on extended-chain crystal formation where modified crystallite dimensions perpendicular to these lattice planes manifest enhanced molecular ordering [28]. To estimate the lamellar thickness of PBS-ECCs, crystallite sizes crystallite sizes *L*_(020)_ and *L*_(021)_ were calculated as 9.4 nm and 11.1 nm using the Debye–Scherrer equation (Figure S3). Based on the known lattice parameters [28,42] and geometric considerations in the *bc* plane [28], the lamellar thickness along the *c*-axis was deduced to exceed 24.1 nm, which is significantly surpasses that of typical folded-chain PBS (<5 nm).

Most significantly, the WAXD patterns of PBS/ECCs composites maintain the characteristic diffraction of the PBS *α*-form crystal structure, with no obviously shifts in peak positions or emergence of new reflections. This structural preservation indicates that ECC incorporation does not disrupt the intrinsic crystalline structure of the PBS matrix. The exceptional crystallographic matching between ECCs and PBS matrix can be attributed to their identical molecular constitution and crystallographic parameters, enabling ECC domains to serve as epitaxial templates for PBS crystallization [44]. This structural compatibility provides significant nucleation advantages compared to conventional nucleating agents [11,45], which typically lack precise lattice correspondence with the polymer matrix.

**Figure 2:**
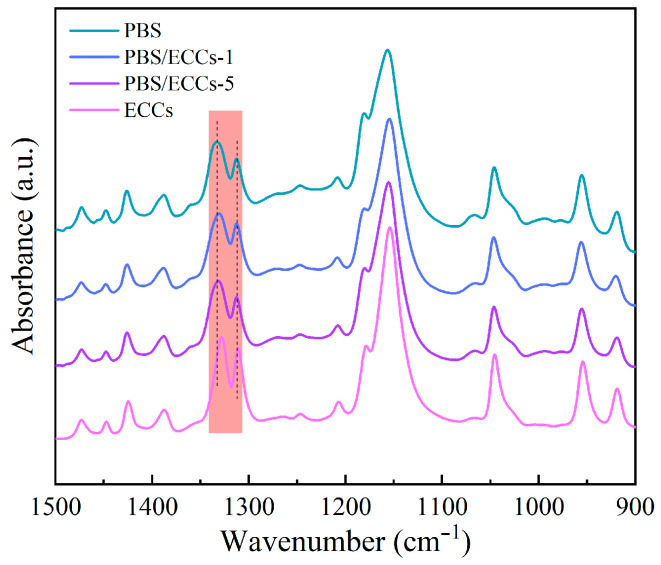
FTIR spectra of PBS and PBS/ECCs composites collected at 25 °C. The color highlighting is necessary to indicate the region of spectral differences.

To gain deeper insights into the molecular structural characteristics and chain conformation, we conducted comprehensive FTIR spectroscopic analysis of PBS, PBS/ECCs composites, and pure ECCs (Figure 2). While the overall spectral patterns in the fingerprint region (1500–900 cm^−1^) remain similar across all samples, detailed examination (marked in red) reveals characteristic variations indicative of conformational transitions. The CH_2_ deformation vibration bands exhibit shifts from 1333 cm^−1^ and 1314 cm^−1^ in PBS shifted to 1329 cm^−1^ and 1312 cm^−1^ in ECCs, respectively [28]. These red-shifts can be attributed to the transition from folded-chain to extended-chain conformations, consistent with previous observations in polyoxymethylene and other semicrystalline polymers [46,47]. Additionally, ECCs exhibited sharper and more well-defined absorption bands compared to neat PBS, reflecting its higher degree of molecular order in the extended-chain structure [48,49]. The FTIR spectra of PBS/ECCs composites exhibit characteristic absorption bands identical to those of the neat PBS matrix, providing complementary evidence for the structural compatibility revealed by XRD analysis [50].

### 3.2. Crystallization Behavior and Thermal Properties

**Figure 3:**
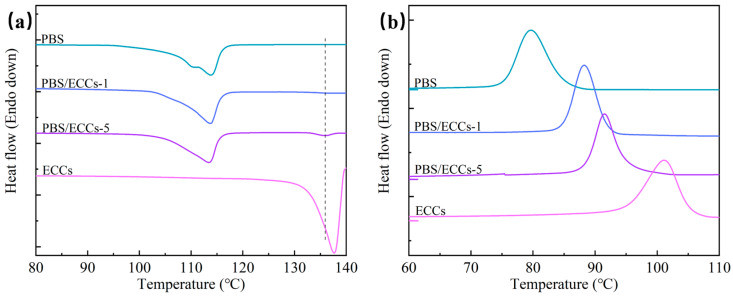
DSC thermograms of PBS, PBS/ECCs-1, PBS/ECCs-5, and ECCs during the first heating (**a**) and the subsequent cooling (**b**) processes at a constant rate of 10 °C/min.

The thermal properties of PBS and PBS/ECC composites were systematically investigated using differential scanning calorimetry (DSC), with the thermograms shown in Figure 3 and the corresponding thermal parameters summarized in Table 1. During the first heating scan (Figure 3a), neat PBS exhibited characteristic multiple melting behavior with overlapping endothermic peaks around 113.5 °C and a total melting enthalpy (Δ*H*_m_) of 61.5 J/g. This multiple melting phenomenon is commonly observed in PBS and can be attributed to the melting–recrystallization–remelting process of crystals with different perfection levels [51,52,53]. Upon incorporation of ECCs, both PBS/ECCs-1 and PBS/ECCs-5 demonstrated enhanced thermal properties. PBS/ECCs-1 showed a slightly higher melting temperature (113.7 °C) with a Δ*H*_m_ of 68.6 J/g. Most notably, PBS/ECCs-5 exhibited two distinct melting endotherms: a primary peak at 113.5 °C (Δ*H*_m_ = 66.3 J/g) corresponding to the melting of the PBS matrix crystals, and a high-temperature peak at 135.9 °C (Δ*H*_m_ = 2.6 J/g) attributed to the melting of preserved ECC structures [28]. This high-temperature melting transition provides direct evidence for the thermal stability of extended-chain crystal structures, further supported by the sharp melting peak of pure ECCs at 137.6 °C with a substantial melting enthalpy of 90.0 J/g [27].

The subsequent cooling scans (Figure 3b) revealed a systematic enhancement in crystallization behavior with increasing ECC content. The crystallization temperature (*T*_c_) progressively increased from 79.6 °C for neat PBS to 88.2 °C for PBS/ECCs-1, and further to 91.5 °C for PBS/ECCs-5, while pure ECCs exhibited the highest *T*_c_ at 101.1 °C. This remarkable increase in *T*_c_ (Δ*T*_c_ = 11.9 °C at 5 wt% loading) demonstrates the exceptional nucleating efficiency of ECCs. This promotion effect will be maintained during multiple thermal cycles (Figure S4), demonstrating the stability of nucleation ability. The enhancement can be attributed to the epitaxial nucleation effect, where the structurally matched ECCs surfaces provide energetically favorable templates that reduce the nucleation barrier for PBS crystallization [27].

**Figure 4:**
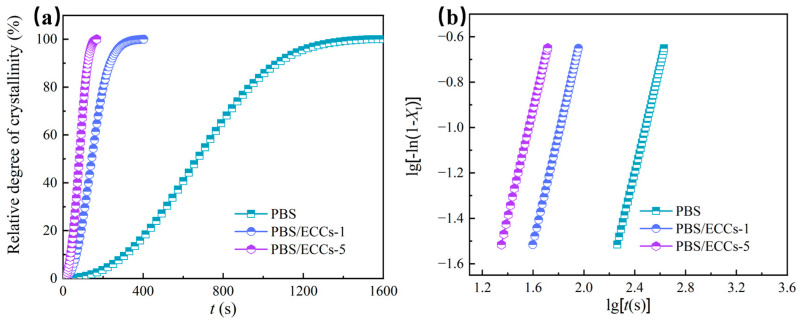
Relative crystallinity curves (**a**) and Avrami plots (**b**) of PBS, PBS/ECCs-1, and PBS/ECCs-5 isothermally crystallized at 98 °C.

To quantitatively evaluate the nucleation efficiency of ECCs, we conducted detailed isothermal crystallization studies at 98 °C. Figure 4a presents the evolution of relative crystallinity (*X_t_*) *versus* time for samples crystallized isothermally at 98 °C. All the samples exhibited characteristic sigmoidal curves, indicating a typical primary crystallization process involving nucleation and subsequent crystal growth [54]. The crystallization rate is dramatically accelerated with increasing ECCs content, manifested by the systematic leftward shift of crystallization curves. The crystallization half-time (*t*_1/2_) decreases markedly from 672 s for neat PBS to 143 s for PBS/ECCs-1, and further to 81 s for PBS/ECCs-5, representing an extraordinary 88% reduction in crystallization time.

The classical Avrami equation [55] (Equation (1)) and the logarithmic form (Equation (2)) here were employed to analyze the isothermal crystallization kinetics. The plots of Equation (2) give *n* as slope and intercept ln*K*, and the resulting *n* and *K* values are listed in Table 2.

(1) $$1-X_{t}=exp(-Kt^{n})$$

(2) $$\mathrm{lg}\left[-\ln\left(1-X_{t}\right)\right]=lgK+nlgt$$

where *n* represents the Avrami exponent reflecting the nucleation mechanism and crystal growth geometry, *t* is the crystallization time and *K* denotes the overall crystallization rate constant.

The plots of lg[−ln(1 − *X*_t_)] versus lg*t* (Figure 4b) exhibited excellent linearity (*R*^2^ > 0.9998) within the relative crystallinity range of 3–20%, indicating that the crystallization process follows classical nucleation and growth mechanism [54]. The Avrami exponent (*n*) remained consistently around 2.4 across all samples, indicating that the incorporation of ECCs primarily influences the nucleation rate rather than altering the crystal growth geometry [11]. This *n* value indicates heterogeneous nucleation mechanism with three-dimensional spherulitic growth, which is typical for PBS crystallization [7,11]. The slight deviation from *n* = 3 (theoretical value for three-dimensional spherulitic growth) can be attributed to the presence of some two-dimensional growth modes, particularly in the early stages of crystallization [56]. The crystallization rate constant (*K*) increased by several orders of magnitude upon ECC addition (from 1.47 × 10^−7^ s^−n^ for neat PBS to 1.95 × 10^−5^ s^−n^ for PBS/ECCs-5), quantitatively confirming the enhanced nucleation efficiency. This dramatic increase in *K* can be attributed to the large number of ECC crystal nuclei and epitaxial nucleation effect of ECCs.

**Figure 5:**
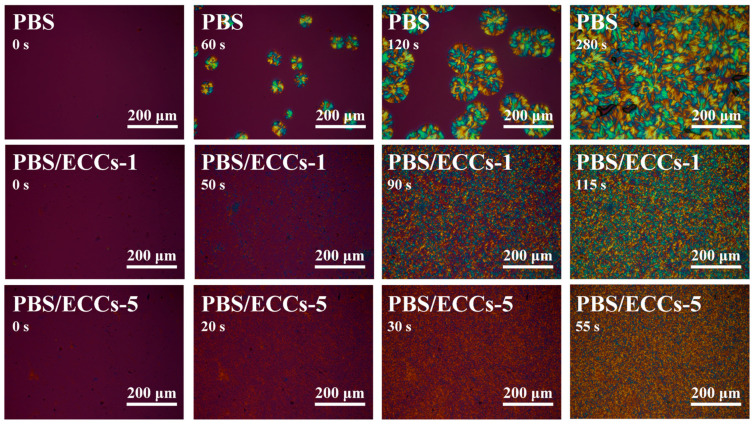
POM images for the crystalline structure variation of PBS, PBS/ECCs-1, and PBS/ECCs-5 isothermally crystallized at 98 °C.

The evolution of the crystalline structure during isothermal crystallization at 98 °C observed by POM is shown in Figure 5. It can be seen that the spherulite size decreases significantly and the nucleation density increases markedly upon the incorporation of ECCs. The visual field of neat PBS exhibited limited nucleation events with sparse spherulite formation, requiring approximately 280 s for complete crystallization. In contrast, both PBS/ECCs-1 and PBS/ECCs-5 composites demonstrated substantially enhanced nucleation efficiency, forming numerous spherulites that filled the observation field within 115 and 55 s, respectively. The markedly increased nucleation density provides direct morphological evidence for the efficient nucleating capability of ECCs [1,7]. This microstructural refinement, characterized by smaller and more uniform spherulites, not only accelerates the overall crystallization process but also suggests potential improvements in mechanical properties of the final materials [57].

### 3.3. Temperature-Dependent Melt Memory Effects

**Figure 6:**
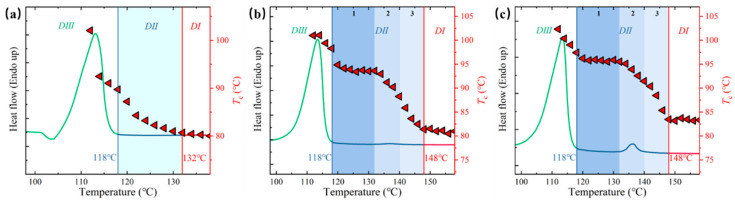
Representation of the self-nucleation domains for PBS (**a**), PBS/ECCs-1 (**b**), and PBS/ECCs-5 (**c**) superimposed on top of the standard DSC melting trace. Data points represent crystallization temperature peaks (right-hand side *y*-axis) as a function of *T*_s_ values (on the *x*-axis). The vertical blue and red lines mark the dividing temperatures between different domains.

The influence of ECCs on melt memory effects was systematically investigated through self-nucleation studies [36,58,59], revealing unprecedented insights into the temperature-dependent crystallization behavior. Analysis of crystallization temperature (*T*_c_) as a function of self-nucleation temperature (*T*_s_) demonstrates an exceptional expansion of *Domain II* (∆*T* = 30 °C, spanning 118–148 °C), significantly broader than that observed in neat PBS (∆*T* = 14 °C) (Figure 6). The DSC cooling curves after isothermal treatment at different *T*_s_ and their subsequent heating scans provide complementary evidence for the domain assignments (Figure S5).

Neat PBS exhibits a conventional self-nucleation profile with a relatively narrow *Domain II* temperature window of 14 °C (118–132 °C) in Figure 6a. While DSC suggests melting of bulk crystals as the temperature increases to 118–132 °C, PBS maintains enhanced nucleation capability through preserved local ordered structure (melt memory effect). This behavior aligns with classical self-nucleation mechanisms observed in semicrystalline polymers [38,60].

**Figure 7:**
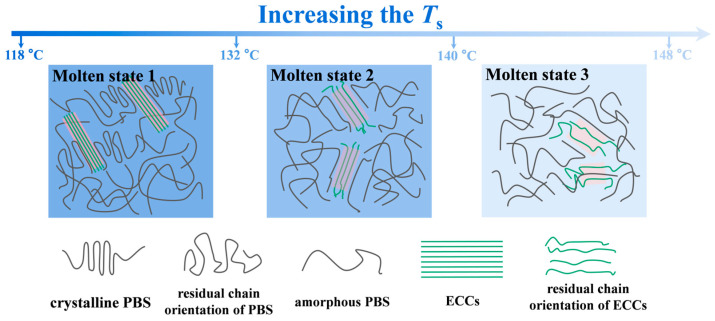
Schematic illustration of the structure evolution of the PBS/ECCs composites melt at different *T*_s_.

The systematic investigation of *T*_c_ as a function of *T*_s_ reveals three distinct stages of the left ordered structures at *T*_s_ (Figure 6b,c and Figure 7). As the temperature increases to 118–132 °C (Molten state 1), we observe a transition where PBS exists in a partially ordered conformational state while ECC crystals remain intact. Despite the loss of PBS crystallites, PBS/ECC composites maintains high crystallization temperatures (95–97 °C) through the combined effect of PBS melt memory and ECC heterogeneous nucleation. Stage 2 (132–140 °C) marks a critical transition point where PBS completely loses its self-nucleation capability, yet the PBS/ECC composites maintain enhanced crystallization capability (91–95 °C) through the exclusive action of remaining ECCs. The preservation of nucleation efficiency in this regime, well above the PBS self-nucleation limit (132 °C) but below the ECCs melting point (138 °C), suggests the unique interactions between ECCs and residual chain orientation of PBS matrix [32,61]. Here, ECCs constrains PBS chain mobility and promotes oriented crystallization [36,58]. Most remarkably, in stage 3 (above 140 °C), even after exceeding the melting point of both components, the PBS/ECC composites continue to exhibit elevated crystallization temperatures (83–91 °C), which is attributed to the melt memory of ECCs (Figure S6).

This comprehensive analysis reveals that ECCs introduce a new paradigm of nucleation control in PBS, characterized by multiple cooperative mechanisms operating across a wide temperature window (∆*T* = 30 °C).

### 3.4. Rheological Analysis of Melt Structure Evolution

**Figure 8:**
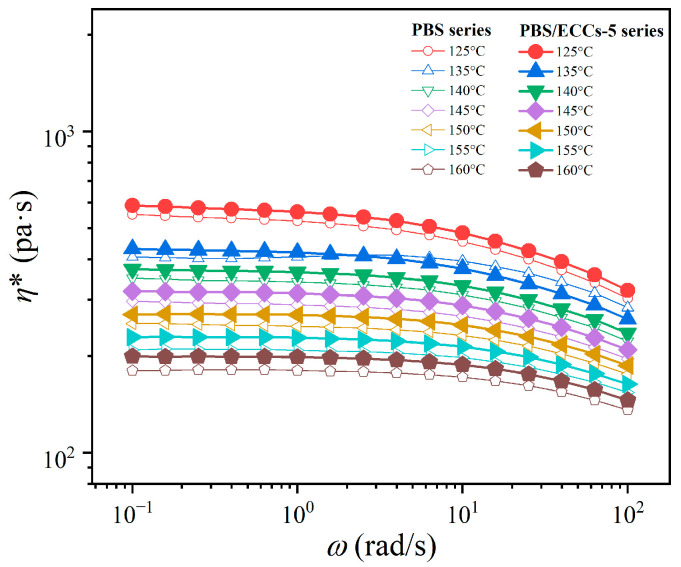
Variation of complex viscosity (|η*|) as functions of frequency for PBS and PBS/ECCs-5 at different temperatures.

Comprehensive rheological investigations were conducted to elucidate the structural evolution and thermal stability of the PBS/ECCs system across a wide temperature range. The complex viscosity measurements (|η*|) across 125 °C to 160 °C (Figure 8) demonstrate remarkable enhancement in viscoelastic response upon ECCs incorporation. This enhancement in viscoelastic response, observed regardless of temperature, indicating significant modifications to the melt structure.

**Figure 9:**
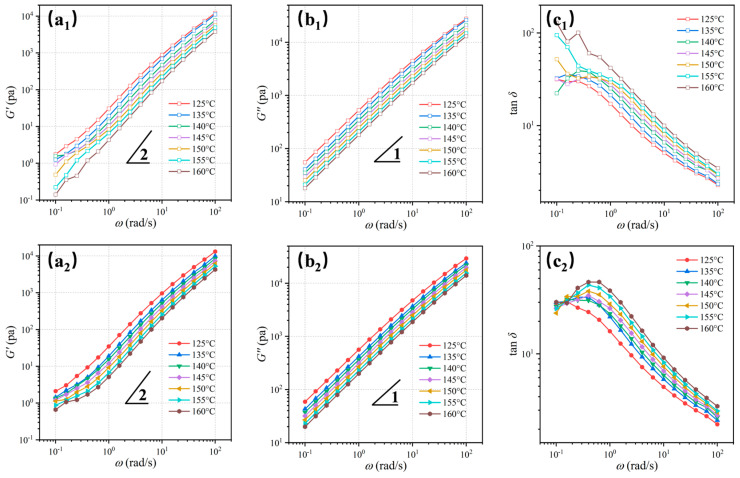
Variation of storage modulus *G*′ (**a_1_**,**a_2_**), loss modulus *G*″ (**b_1_**,**b_2_**), and loss tangent (tan *δ*) (**c_1_**,**c_2_**) as functions of frequency for PBS and PBS/ECCs-5 at different temperatures.

The frequency dependences of storage modulus (*G*′), loss modulus (*G*″), and loss tangent (tan *δ* = *G*″/*G*′) measurements for PBS and PBS/ECCs-5 provide detailed insights into the melting characteristics (Figure 9). Neat PBS exhibits classical terminal viscoelastic behavior typical of homogeneous polymer melts [62], characterized by *G*′∝*ω*^2^ and *G*″∝*ω*^1^ scaling relationships at low frequencies (Figure 9(a_1_,b_1_)). In contrast, PBS/ECCs-5 shows significant deviation from terminal behavior, manifested by a reduction in the power-law dependence of *G*’ to approximately *ω*^0.8^ in the terminal region (Figure 9(a_2_)). This non-terminal response, coupled with systematically elevated *G*″ values across all investigated temperatures (Figure 9(b_2_)), provides compelling evidence for the emergence of slow relaxation processes and the enhanced melt elasticity characteristic of network structures [57,61,63,64,65].

The loss tangent (tan *δ*) serves as a highly sensitive indicator of viscoelastic behavior, providing more nuanced insights into relaxation phenomena than G′ and G″, offering nuanced insights into molecular mobility and structural constraints [66]. As shown in Figure 9(c_1_), neat PBS displays characteristic viscoelastic liquid behavior, with tan *δ* values decreasing systematically with increasing frequency, reflecting unrestricted chain mobility typical of homogeneous polymer melts. Below 135 °C, a moderate reduction in tan *δ* values appears in the low-frequency region, attributable to residual chain ordered structure [36]. The most significant findings emerge at elevated temperatures (150 °C and 160 °C), where both PBS matrix (folded-chain crystals FCCs) and ECC structures have nominally melted. Under these conditions, PBS/ECCs-5 maintains reduced tan *δ* values compared to neat PBS, particularly pronounced in the low-frequency regime (Figure 9(c_2_)). This unusual viscoelastic response indicates the persistence of specific chain–chain interactions between PBS and ECCs components. Relaxation modulus *G*(*t*) conducted at various temperatures (Figure S7) provide complementary evidence for the structural characteristics [67,68], showing higher relaxation modulus for PBS/ECCs-5 compared to neat PBS, particularly at lower temperatures and longer relaxation times.

To provide definitive evidence of the distinctive structure present within the polymer melt, rheological investigations were extended to PBS/ECCs-10 composites with increased ECCs concentration. This higher loading condition exhibits more pronounced rheological responses that enable a precise characterization of the viscoelastic behavior. The complex viscosity measurements of PBS/ECCs-10 exhibit significantly elevated values compared to neat PBS (Figure S8), demonstrating a systematic enhancement in viscoelastic response.

**Figure 10:**
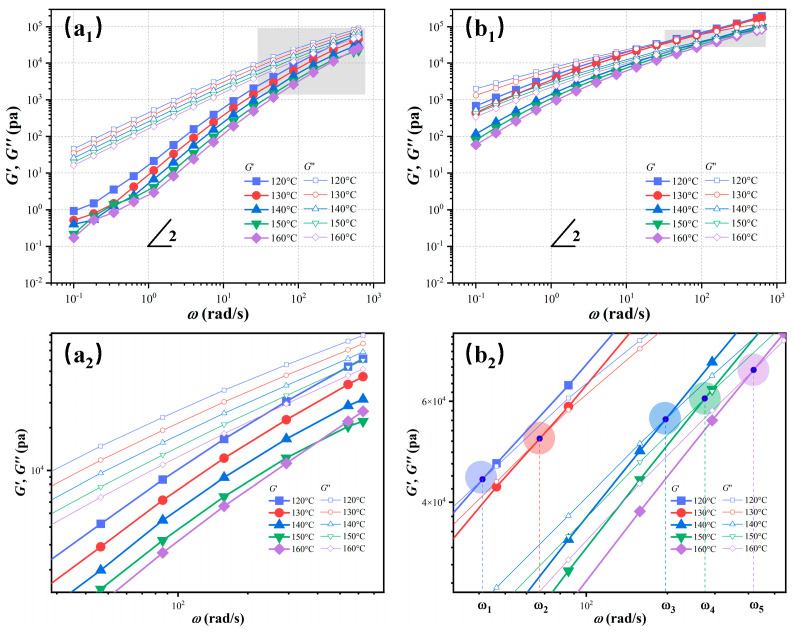
Variation of storage modulus *G*′ and loss modulus *G*″ of PBS (**a_1_**,**a_2_**) and PBS/ECCs-10 (**b_1_**,**b_2_**) as functions of frequency at different temperatures, where **a_2_** and **b_2_** show magnified views of selected regions from **a_1_** and **b_1_**, respectively.

Figure 10 presents the frequency-dependent viscoelastic response of PBS and PBS/ECCs-10 at different temperatures. Neat PBS exhibits classical homopolymer terminal behavior indicating complete chain relaxation in the melt state (Figure 10(a_1_)) [62]. In contrast, the PBS/ECCs-10 system exhibits a pronounced non-terminal response (Figure 10(b_1_)). This behavior signifies incomplete chain relaxation and provides strong evidence for the presence of the physical network structure [69]. The most significant rheological signature observed is the distinct crossover phenomenon between G′ and G″ in PBS/ECCs-10, which is notably absent in neat PBS within the measured frequency range. This crossover point, where *G*′ = *G*″, defines the characteristic relaxation time (*τ*) according to the relation *τ* = 1/*ω*_cr_, where *ω*_cr_ is the crossover frequency [70,71].

Quantitative analysis reveals a systematic temperature dependence of the crossover frequencies (*ω*_cr_) in PBS/ECCs-10, with specific values of *ω*_1_ = 41 rad/s, *ω*_2_ = 67 rad/s, *ω*_3_ = 197 rad/s, *ω*_4_ = 274 rad/s, and *ω*_5_ = 419 rad/s measured at 120 °C, 130 °C, 140 °C, 150 °C, and 160 °C, respectively (Figure 10(b_2_)). These correspond to the average relaxation times (τ = 1/*ω*_cr_) of 0.024, 0.015, 0.005, 0.004, and 0.002 s, exhibiting a systematic decrease with increasing temperature. Conversely, neat PBS fails to display such crossover behavior within the accessible frequency window (Figure 10(b_1_)), indicating substantially shorter relaxation times typical of homopolymer melts. This fundamental difference in relaxation behavior underscores the profound structural modifications induced by the incorporation of ECCs. Most significantly, a well-defined crossover point persists even at 160 °C (~20 °C above the *T*_m_ of ECCs). This observation confirms the exceptional thermal stability of the intermolecular interactions and provides a rheological basis for the enhanced melt memory effects observed in the self-nucleation studies.

## 4. Conclusions

In this work, we have systematically investigated the exceptional efficacy of PBS-ECC as a nucleating agent for poly(butylene succinate). Our comprehensive analyses reveal that the incorporation of ECCs induces remarkable enhancement in crystallization kinetics, evidenced by an 11.9 °C increase in crystallization temperature and an 88% reduction in crystallization half-time at 5 wt% loading, stemming from the unique structural compatibility between ECCs and the PBS matrix. Most significantly, self-nucleation studies revealed an unprecedented expansion of *Domain II* (∆*T* = 30 °C), characterizing distinct melt memory effects across a wider temperature range, which substantially broadens the processing window for industrial applications. Rheological investigations further elucidated the formation of a thermally stable local ordered structure maintained through specific chain recognition mechanisms. The non-terminal viscoelastic response, evidenced by reduced power-law dependence of storage modulus and systematically decreased loss tangent values, persisted even at temperatures exceeding the nominal melting points of both PBS matrix and ECCs. Complementary analyses of PBS/ECCs-10 composites provided definitive evidence for the existence of unique structures in the melt, characterized by distinct *G*′-*G*″ crossover phenomena with systematic temperature dependence of relaxation times. These findings establish new fundamental insights into nucleation control in biodegradable polyesters while offering practical strategies for developing high-performance sustainable materials.

## Figures and Tables

**Table 1:** Thermal parameters of PBS, PBS/ECCs-1, PBS/ECCs-5, and ECCs.

Sample	*T*_c_ (°C)	Δ*H*_c_ (J/g)	*T*_m_ (°C)	Δ*H*_m_ (J/g)
PBS	79.6	66.8	113.5	61.5
PBS/ECCs-1	88.3	63.4	113.7	68.6
PBS/ECCs-5	91.5	60.5	113.5/135.9	66.3/2.6
ECCs	101.1	79.7	137.6	90.0

The crystallization temperature (*T*_c_), melting temperature (*T*_m_), and enthalpy (Δ*H*) obtained from Figure 3.

**Table 2:** Avrami parameters of PBS, PBS/ECCs-1, and PBS/ECCs-5 isothermally crystallized at 98 °C.

Samples	*n*	*K* (s^−*n*^)	*t*_1/2_ (s)	*R* ^2^
PBS	2.4	1.47 × 10^−7^	672	0.99988
PBS/ECCs-1	2.4	4.24 × 10^−6^	143	0.99995
PBS/ECCs-5	2.4	1.95 × 10^−5^	81	0.99999

## Data Availability

The original contributions presented in this study are included in the article/Supplementary Material. Further inquiries can be directed to the corresponding authors.

## Supplementary Materials

The following supporting information can be downloaded at: https://www.mdpi.com/article/10.3390/polym17081086/s1, Figure S1: Scheme of a self-nucleation (SN) experiment, Figure S2: Strain amplitude dependence of storage modulus (G′), loss modulus (G″), and shear stress (τ) for PBS, Figure S3: Intensities and fits of wide-angle X-ray diffractogram of PBS-ECC, Figure S4: DSC thermograms of PBS/ECCs-5 during the multiple thermal cycles at a constant rate of 10 °C/min, Figure S5: Self-nucleation of PBS, PBS/ECCs-1, PBS/ECCs-5 (a,b,c) DSC cooling scans from the indicated *T*_s_ and the subsequent heating scans (d,e,f), Figure S6: Representation of the self-nucleation domains for ECCs superimposed on top of the standard DSC melting trace. Data points represent crystallization temperature peaks (right-hand side *y*-axis) as a function of Ts values (on the *x*-axis). The vertical blue and red lines mark the dividing temperatures between different domains, Figure S7: Relaxation modulus *G*(*t*) of PBS and PBS/ECCs-5 at different temperatures. The amplitude of the strain before relaxation was 10%, Figure S8: Variation of complex viscosity (|η*|) as functions of frequency for PBS and PBS/ECCs-10 at different temperatures.

## References

[1] Xu J., Guo B.H. (2010). Poly(butylene succinate) and its copolymers: Research, development and industrialization. Biotechnol. J..

[2] Ray S.S., Okamoto M. (2003). Biodegradable polylactide and its nanocomposites: Opening a new dimension for plastics and composites. Macromol. Rapid Commun..

[3] Pérez-Camargo R.A., Liu G., Cavallo D., Wang D., Müller A.J. (2020). Effect of the Crystallization Conditions on the Exclusion/Inclusion Balance in Biodegradable Poly(butylene succinate-*ran*-butylene adipate) Copolymers. Biomacromolecules.

[4] Gigli M., Fabbri M., Lotti N., Gamberini R., Rimini B., Munari A. (2016). Poly(butylene succinate)-based polyesters for biomedical applications: A review. Eur. Polym. J..

[5] Barletta M., Aversa C., Ayyoob M., Gisario A., Hamad K., Mehrpouya M., Vahabi H. (2022). Poly(butylene succinate)(PBS): Materials, processing, and industrial applications. Prog. Polym. Sci..

[6] Li Y., Huang G., Chen C., Wei X.W., Dong X., Zhao W., Ye H.M. (2021). Poly(butylene succinate-*co*-butylene acetylenedicarboxylate): Copolyester with Novel Nucleation Behavior. Polymers.

[7] Papageorgiou G.Z., Achilias D.S., Bikiaris D.N. (2007). Crystallization kinetics of biodegradable poly(butylene succinate) under isothermal and non-isothermal conditions. Macromol. Chem. Phys..

[8] Gan Z., Abe H., Kurokawa H., Doi Y. (2001). Solid-state microstructures, thermal properties, and crystallization of biodegradable poly(butylene succinate) (PBS) and its copolyesters. Biomacromolecules.

[9] Zheng Y., Pan P. (2020). Crystallization of biodegradable and biobased polyesters: Polymorphism, cocrystallization, and structure-property relationship. Prog. Polym. Sci..

[10] Fujimaki T. (1998). Processability and properties of aliphatic polyesters, ‘BIONOLLE’, synthesized by polycondensation reaction. Polym. Degrad. Stab..

[11] Tan L., Chen Y., Zhou W., Ye S., Wei J. (2011). Novel approach toward poly(butylene succinate)/single-walled carbon nanotubes nanocomposites with interfacial-induced crystallization behaviors and mechanical strength. Polymer.

[12] Bian J., Han L., Wang X., Wen X., Han C., Wang S., Dong L. (2010). Nonisothermal crystallization behavior and mechanical properties of poly(butylene succinate)/silica nanocomposites. J. Appl. Polym. Sci..

[13] Jin T.-X., Liu C., Zhou M., Chai S.-g., Chen F., Fu Q. (2015). Crystallization, mechanical performance and hydrolytic degradation of poly(butylene succinate)/graphene oxide nanocomposites obtained via in situ polymerization. Compos. Part A Appl. Sci. Manuf..

[14] Shih Y.-F., Chen L., Jeng R. (2008). Preparation and properties of biodegradable PBS/multi-walled carbon nanotube nanocomposites. Polymer.

[15] Li J., Luo X., Lin X. (2013). Preparation and characterization of hollow glass microsphere reinforced poly(butylene succinate) composites. Mater. Des..

[16] Kuilla T., Bhadra S., Yao D., Kim N.H., Bose S., Lee J.H. (2010). Recent advances in graphene based polymer composites. Prog. Polym. Sci..

[17] Wittmann J.C., Lotz B. (1981). Epitaxial crystallization of polyethylene on organic substrates: A reappraisal of the mode of action of selected nucleating agents. J. Polym. Sci. Polym. Phys. Ed..

[18] Bledsoe J.C., Crane G.H., Locklin J.J. (2023). Beyond Lattice Matching: The Role of Hydrogen Bonding in Epitaxial Nucleation of Poly(hydroxyalkanoates) by Methylxanthines. ACS Appl. Polym. Mater..

[19] Liu L., Zhao Y., Zhang C., Dong Z., Wang K., Wang D. (2021). Morphological Characteristics of *β*-Nucleating Agents Governing the Formation of the Crystalline Structure of Isotactic Polypropylene. Macromolecules.

[20] Bai H., Zhang W., Deng H., Zhang Q., Fu Q. (2011). Control of Crystal Morphology in Poly(*l*-lactide) by Adding Nucleating Agent. Macromolecules.

[21] Ye H.-M., Wang R.-D., Liu J., Xu J., Guo B.-H. (2012). Isomorphism in poly(butylene succinate-*co*-butylene fumarate) and its application as polymeric nucleating agent for poly(butylene succinate). Macromolecules.

[22] Ye H.-M., Tang Y.-R., Xu J., Guo B.-H. (2013). Role of poly(butylene fumarate) on crystallization behavior of poly(butylene succinate). Ind. Eng. Chem. Res..

[23] Wei X.-W., Yang L.-L., Li Y., Meng X., Cai L.-H., Zhou Q., Ye H.-M. (2021). Asymmetrical formation of isomorphism in the crystalline/crystalline blend of poly(butylene succinate) and poly(butylene fumarate). Polymer.

[24] Zheng Y., Tian G., Xue J., Zhou J., Huo H., Li L. (2018). Effects of isomorphic poly(butylene succinate-*co*-butylene fumarate) on the nucleation of poly(butylene succinate) and the formation of poly(butylene succinate) ring-banded spherulites. CrystEngComm.

[25] Varga J., Karger-Kocsis J. (1996). Rules of supermolecular structure formation in sheared isotactic polypropylene melts. J. Polym. Sci. Part B Polym. Phys..

[26] Xu J., Ma Y., Hu W., Rehahn M., Reiter G. (2009). Cloning polymer single crystals through self-seeding. Nat. Mater..

[27] Ye H.-M., Chen X.-T., Li H.-F., Zhang P., Ma W., Li B., Xu J. (2019). Industrializable and sustainable approach for preparing extended-chain crystals of biodegradable poly(butylene succinate) and their applications. Polymer.

[28] Ye H.-M., Chen X.-T., Liu P., Wu S.-Y., Jiang Z., Xiong B., Xu J. (2017). Preparation of poly(butylene succinate) crystals with exceptionally high melting point and crystallinity from its inclusion complex. Macromolecules.

[29] Brochu S., Prud’homme R.E., Barakat I., Jerome R. (1995). Stereocomplexation and Morphology of Polylactides. Macromolecules.

[30] Yamane H., Sasai K. (2003). Effect of the addition of poly(*D*-lactic acid) on the thermal property of poly(*L*-lactic acid). Polymer.

[31] Rahman N., Kawai T., Matsuba G., Nishida K., Kanaya T., Watanabe H., Okamoto H., Kato M., Usuki A., Matsuda M. (2009). Effect of Polylactide Stereocomplex on the Crystallization Behavior of Poly(*L*-lactic acid). Macromolecules.

[32] Wei X.-F., Bao R.-Y., Cao Z.-Q., Yang W., Xie B.-H., Yang M.-B. (2014). Stereocomplex Crystallite Network in Asymmetric PLLA/PDLA Blends: Formation, Structure, and Confining Effect on the Crystallization Rate of Homocrystallites. Macromolecules.

[33] Schmidt S.C., Hillmyer M.A. (2001). Polylactide stereocomplex crystallites as nucleating agents for isotactic polylactide. J. Polym. Sci. Part B Polym. Phys..

[34] Yang L.-L., Wei X.-W., Wu T., Ye H.-M. (2024). Crystallization manipulation of poly(butylene succinate) via hydrazide-based self-assembled hydrogen-bonding interactions. Polymer.

[35] Reid B.O., Vadlamudi M., Mamun A., Janani H., Gao H., Hu W., Alamo R.G. (2013). Strong memory effect of crystallization above the equilibrium melting point of random copolymers. Macromolecules.

[36] Sangroniz L., Cavallo D., Müller A.J. (2020). Self-Nucleation Effects on Polymer Crystallization. Macromolecules.

[37] Zhang D., Zhao R.-J., Ma G.-Q., Ma Z. (2023). Self-nucleation Effect of Crystallization with Various Nucleating Agents. Chin. J. Polym. Sci..

[38] Fillon B., Wittmann J., Lotz B., Thierry A. (1993). Self-nucleation and recrystallization of isotactic polypropylene (*α* phase) investigated by differential scanning calorimetry. J. Polym. Sci. Part B Polym. Phys..

[39] Solomon O., Ciutǎ I. (1962). Détermination de la viscosité intrinsèque de solutions de polymères par une simple détermination de la viscosité. J. Appl. Polym. Sci..

[40] Maghami G.G., Roberts G.A. (1988). Evaluation of the viscometric constants for chitosan. Die Makromol. Chem. Macromol. Chem. Phys..

[41] Yang L.-L., Wei X.-W., Wu T., Meng X., Ye H.-M. (2023). Manipulating the Co-crystallization Behavior in Isomorphic Copolymers by Introducing a Third Monomeric Unit. Macromolecules.

[42] Ihn K., Yoo E., Im S. (1995). Structure and morphology of poly(tetramethylene succinate) crystals. Macromolecules.

[43] Ichikawa Y., Kondo H., Igarashi Y., Noguchi K., Okuyama K., Washiyama J. (2000). Crystal structures of *α* and *β* forms of poly(tetramethylene succinate). Polymer.

[44] Binsbergen F. (1970). Heterogeneous nucleation in the crystallization of polyolefins: Part 1. Chemical and physical nature of nucleating agents. Polymer.

[45] Tang Y.-R., Lin D.-W., Gao Y., Xu J., Guo B.-H. (2014). Prominent nucleating effect of finely dispersed hydroxyl-functional hexagonal boron nitride on biodegradable poly(butylene succinate). Ind. Eng. Chem. Res..

[46] Zhang J., Duan Y., Sato H., Tsuji H., Noda I., Yan S., Ozaki Y. (2005). Crystal modifications and thermal behavior of poly(*L*-lactic acid) revealed by infrared spectroscopy. Macromolecules.

[47] Wei M., Shin I.D., Urban B., Tonelli A.E. (2004). Partial miscibility in a nylon-6/nylon-66 blend coalesced from their common *α*-cyclodextrin inclusion complex. J. Polym. Sci. Part B Polym. Phys..

[48] Kongkhlang T., Tashiro K., Kotaki M., Chirachanchai S. (2008). Electrospinning as a new technique to control the crystal morphology and molecular orientation of polyoxymethylene nanofibers. J. Am. Chem. Soc..

[49] Kobayashi M., Sakashita M., Hasegawa M. (1991). Infrared and Raman spectra of macrocyclic poly(oxymethylene). Macromolecules.

[50] Sangroniz L., Alamo R.G., Cavallo D., Santamaría A., Müller A.J., Alegría A. (2018). Differences between Isotropic and Self-Nucleated PCL Melts Detected by Dielectric Experiments. Macromolecules.

[51] Wang X., Zhou J., Li L. (2007). Multiple melting behavior of poly(butylene succinate). Eur. Polym. J..

[52] Qiu Z., Komura M., Ikehara T., Nishi T. (2003). DSC and TMDSC study of melting behaviour of poly(butylene succinate) and poly(ethylene succinate). Polymer.

[53] Yasuniwa M., Tsubakihara S., Satou T., Iura K. (2005). Multiple melting behavior of poly(butylene succinate). II. Thermal analysis of isothermal crystallization and melting process. J. Polym. Sci. Part B Polym. Phys..

[54] Lorenzo A.T., Arnal M.L., Albuerne J., Müller A.J. (2007). DSC isothermal polymer crystallization kinetics measurements and the use of the Avrami equation to fit the data: Guidelines to avoid common problems. Polym. Test..

[55] Avrami M. (1939). Kinetics of Phase Change. I General Theory. J. Chem. Phys..

[56] Tsuji H., Yamashita Y. (2014). Highly accelerated stereocomplex crystallization by blending star-shaped 4-armed stereo diblock poly(lactide)s with poly(*D*-lactide) and poly(*L*-lactide) cores. Polymer.

[57] Li Y.-D., Fu Q.-Q., Wang M., Zeng J.-B. (2017). Morphology, crystallization and rheological behavior in poly(butylene succinate)/cellulose nanocrystal nanocomposites fabricated by solution coagulation. Carbohydr. Polym..

[58] Liu X., Wang Y., Wang Z., Cavallo D., Müller A.J., Zhu P., Zhao Y., Dong X., Wang D. (2020). The origin of memory effects in the crystallization of polyamides: Role of hydrogen bonding. Polymer.

[59] Lorenzo A.T., Arnal M.L., Sanchez J.J., Müller A.J. (2006). Effect of annealing time on the self-nucleation behavior of semicrystalline polymers. J. Polym. Sci. Part B Polym. Phys..

[60] Pérez R., Córdova M., López J., Hoskins J., Zhang B., Grayson S., Müller A. (2014). Nucleation, crystallization, self-nucleation and thermal fractionation of cyclic and linear poly(*ε*-caprolactone) s. React. Funct. Polym..

[61] Jing Z., Shi X., Zhang G., Li J., Li J., Zhou L., Zhang H. (2016). Formation, structure and promoting crystallization capacity of stereocomplex crystallite network in the poly(lactide) blends based on linear PLLA and PDLA with different structures. Polymer.

[62] Sun Y., Wu L., Bu Z., Li B.-G., Li N., Dai J. (2014). Synthesis and Thermomechanical and Rheological Properties of Biodegradable Long-Chain Branched Poly(butylene succinate-*co*-butylene terephthalate) Copolyesters. Ind. Eng. Chem. Res..

[63] Xu Z., Zhang Y., Wang Z., Sun N., Li H. (2011). Enhancement of electrical conductivity by changing phase morphology for composites consisting of polylactide and poly(*ε*-caprolactone) filled with acid-oxidized multiwalled carbon nanotubes. ACS Appl. Mater. Interfaces.

[64] Liu K., Andablo-Reyes E., Patil N., Merino D.H., Ronca S., Rastogi S. (2016). Influence of reduced graphene oxide on the rheological response and chain orientation on shear deformation of high density polyethylene. Polymer.

[65] Tsuji H., Hyon S.H., Ikada Y. (1991). Stereocomplex formation between enantiomeric poly(lactic acid)s. 3. Calorimetric studies on blend films cast from dilute solution. Macromolecules.

[66] Xu Z., Niu Y., Wang Z., Li H., Yang L., Qiu J., Wang H. (2011). Enhanced nucleation rate of polylactide in composites assisted by surface acid oxidized carbon nanotubes of different aspect ratios. ACS Appl. Mater. Interfaces.

[67] Yamada T., Yamaguchi M., Kida T. (2022). Evaluation of Rheological Properties and Generation of Cross Orientation of Lamellae and Amorphous Chains in a Thermoplastic Polyester Elastomer. ACS Appl. Polym. Mater..

[68] Bai J., Wang J., Wang W., Fang H., Xu Z., Chen X., Wang Z. (2016). Stereocomplex Crystallite-Assisted Shear-Induced Crystallization Kinetics at a High Temperature for Asymmetric Biodegradable PLLA/PDLA Blends. ACS Sustain. Chem. Eng..

[69] Winter H.H. (2013). Glass transition as the rheological inverse of gelation. Macromolecules.

[70] Winter H.H., Chambon F. (1986). Analysis of linear viscoelasticity of a crosslinking polymer at the gel point. J. Rheol..

[71] Elmoumni A., Winter H.H., Waddon A.J., Fruitwala H. (2003). Correlation of material and processing time scales with structure development in isotactic polypropylene crystallization. Macromolecules.

