# Supplementary material for: Enhanced Melt Memory Effects in Poly(butylene succinate) Through Incorporation of Extended-Chain Crystals
Source: Polymers (Basel). 2025 Apr 17;17(8):1086. doi: 10.3390/polym17081086 (PMC12030295; doi:10.3390/polym17081086)
Supplement: Supplementary file 1 [file polymers-17-01086-s001.zip › polymers-3577955-supplementary.pdf]

Supplementary Materials for

Enhanced Melt Memory Effects in Poly(butylene succinate) Through Incorporation of Extended-Chain Crystals

Xue-Wei Wei [1,2], Jun Xu [1], Jia-Yao Chen [2], Bao-Hua Guo [1,*] and Hai-Mu Ye [2,*]

[1] Institute of Polymer Science & Engineering, Department of Chemical Engineering, Tsinghua University, Beijing 100084, China

[2] Department of Materials Science and Engineering, College of New Energy and Materials, China University of Petroleum, Beijing 102249, China

* Correspondence: bhguo@mail.tsinghua.edu.cn (B.-H.G.); yehaimu@cup.edu.cn (H.-M.Y.)

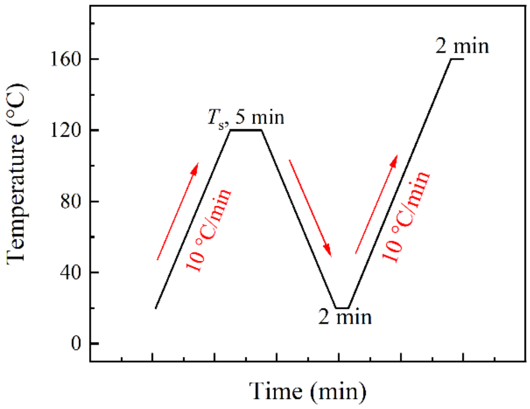

Figure S1. Scheme of a self-nucleation (SN) experiment.

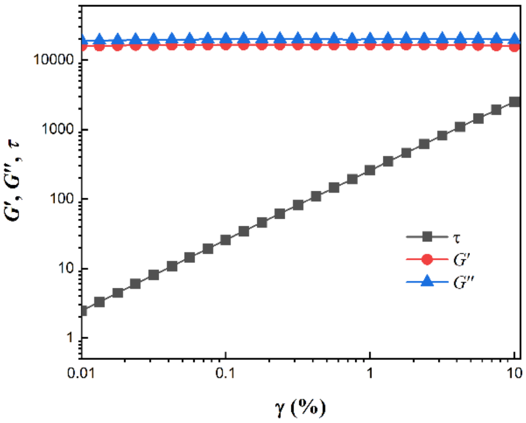

Figure S2. Strain amplitude dependence of storage modulus (G′), loss modulus (G″), and shear stress (τ) for PBS.

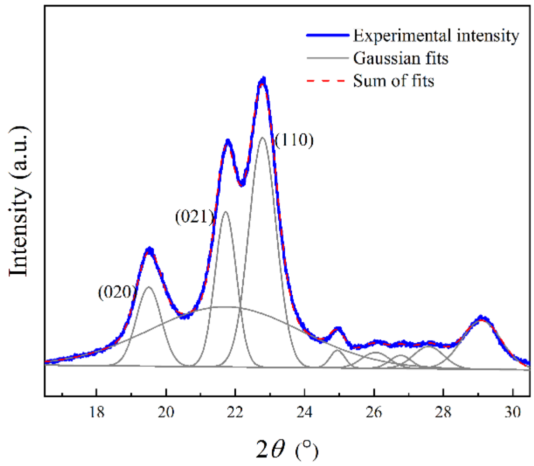

Figure S3. Intensities and fits of wide angle X-ray diffractogram of PBS-ECC.

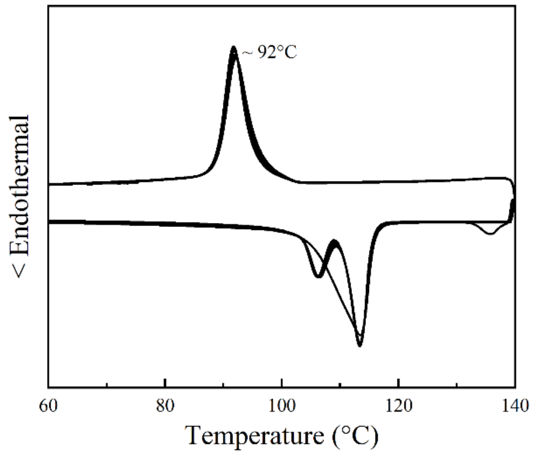

Figure S4. DSC thermograms of PBS/ECCs-5 during the multiple thermal cycles at a constant rate of 10 °C/min.

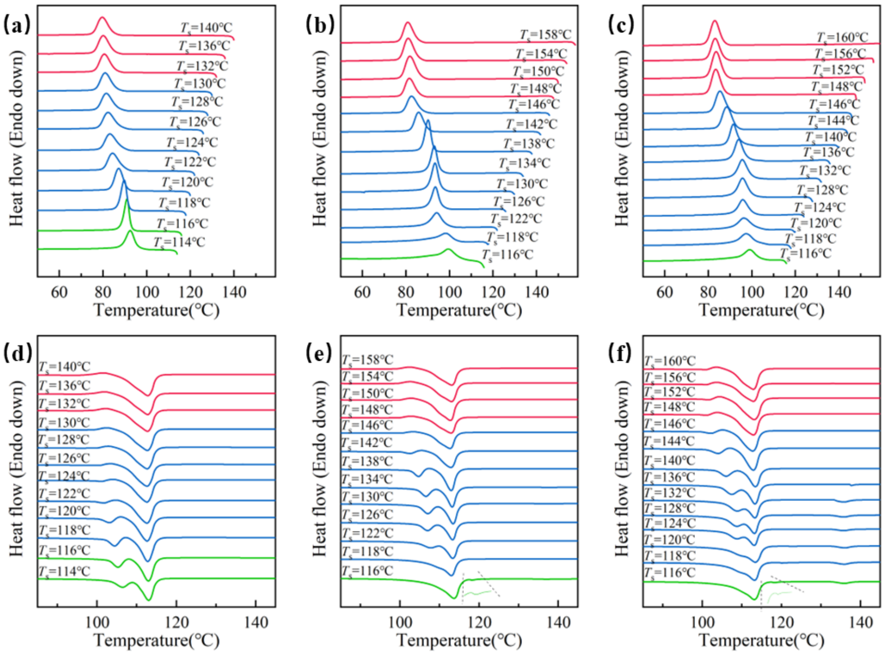

Figure S5. Self-nucleation of PBS, PBS/ECCs-1, PBS/ECCs-5 (a, b, c) DSC cooling scans from the indicated T_s and the subsequent heating scans (d, e, f).

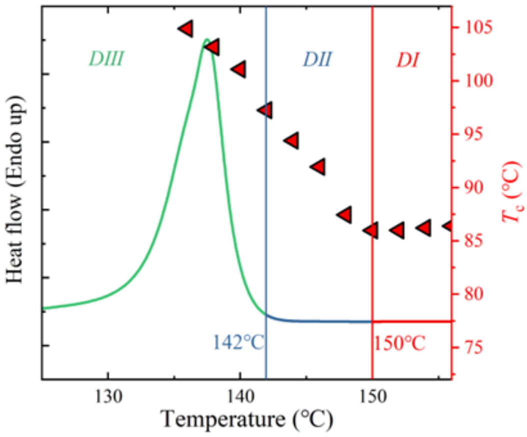

Figure S6. Representation of the self-nucleation domains for ECCs superimposed on top of the standard DSC melting trace. Data points represent crystallization temperature peaks (right-hand side *y*-axis) as a function of T_s values (on the *x*-axis). The vertical blue and red lines mark the dividing temperatures between different domains.

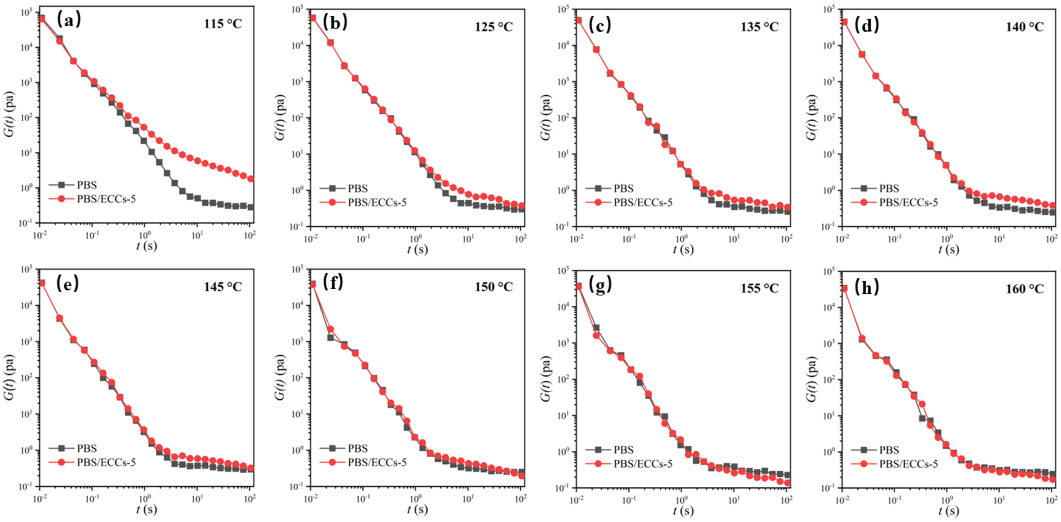

Figure S7. Relaxation modulus G(t) of PBS and PBS/ECCs-5 at different temperatures. The amplitude of the strain before relaxation was 10%.

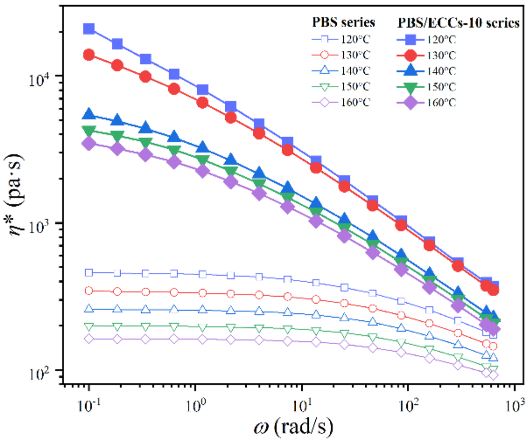

Figure S8. Variation of complex viscosity (|η*|) as functions of frequency for PBS and PBS/ECCs-10 at different temperatures.
